# Structural insights into the activation of USP46 by WDR48 and WDR20

**DOI:** 10.1038/s41421-019-0102-1

**Published:** 2019-07-02

**Authors:** Hanwen Zhu, Tianlong Zhang, Fang Wang, Jun Yang, Jianping Ding

**Affiliations:** State Key Laboratory of Molecular Biology, CAS Center for Excellence in Molecular Cell Science, Shanghai Institute of Biochemistry and Cell Biology, University of Chinese Academy of Sciences, Chinese Academy of Sciences, 320 Yue-Yang Road, Shanghai, 200031 China

**Keywords:** Nanocrystallography, Deubiquitylating enzymes

Dear Editor,

Ubiquitination is an important and reversible post-translational modification that regulates the stability, localization, and function of proteins in many cellular processes. Deubiquitinases (DUBs) are responsible for the removal of ubiquitin chains from proteins, and ubiquitin-specific proteases (USPs) are the largest family of DUBs which share a conserved USP catalytic domain^1^. The USP domain consists of three subdomains named as fingers, palm, and thumb. The catalytic center comprised of a conserved triad (Cys, Asp, and His) is located at the interface of the palm and thumb subdomains, and the fingers subdomain is involved in the binding of Ub. The activity of USPs can be regulated through various ways including, posttranslational modification, allosteric regulation, and interaction of binding partner^2^. A number of WD40-repeat (WDR) proteins have been identified to interact with and regulate the activity of USPs^3^. USP1, USP12, and USP46 constitute a subfamily of USPs which contain a single USP domain and share a common WDR partner WDR48 (also called UAF1) whose binding can stimulate the activity of these USPs^4,5^. Besides, USP12 and USP46 can also bind with WDR20 but USP1 cannot; and the activity of USP12 and USP46 can be activated by WDR48 and WDR20 independently and synergistically^6–8^. This difference is correlated with their different functional roles in distinct biological processes. USP1 is responsible for deubiquitination of proteins mainly involved in DNA damage repairing, whereas USP12 and USP46 for deubiquitination of proteins mainly involved in cell development and cellular signaling^7–11^. So far, the molecular mechanisms for how these USPs recognize their substrates and how the WDR proteins regulate the activities of USPs remain elusive. Recently, the structures of USP46 show that WDR48 binds to the fingers subdomain of USP46 and the binding does not induce notable conformational changes surrounding the catalytic center but may stabilize the substrate binding, and thus potentiates the activity of USP46^12^. However, the structures of USP12 show that WDR48 and WDR20 bind to the fingers and palm subdomains of USP12, respectively, and the binding induces conformational changes surrounding the catalytic center, suggesting that the WDR proteins may modulate the activity of USP12 via allosteric regulation^13,14^.

To further investigate the molecular mechanism of the activation of USP46 by WDR48 and WDR20, we prepared, crystallized and determined the structure of the USP46-WDR48-WDR20 complex at 3.1 Å resolution (Fig. 1a, Supplementary Figs. S1, 2, Table S1). There is one complex per asymmetric unit, in which most residues of the three proteins are well defined except for a few surface exposed residues. The overall structure of the USP46-WDR48-WDR20 complex is very similar to that of the USP12-WDR48-WDR20 complex^13^ (Supplementary Fig. S3). The tip of the fingers subdomain of USP46 is stabilized by a Zn^2+^ coordinated by four conserved Cys residues; and the active site assumes a catalytically conducive conformation^15^. WDR48 and WDR20 bind to the tip of the fingers subdomain and the base of the palm subdomain of USP46, respectively, and are located remotely from the catalytic center (Fig. 1a). As the USP46-WDR48 interface is essentially identical to that in the USP46-WDR48-Ub complex^12^, and the importance of the key residues at this interface has been validated previously^12^, we would not repeat here. The USP46-WDR20 interface is mediated by both hydrophobic and hydrophilic interactions, and involves two loops at the base of the palm subdomain of USP46 and several top surface loops of WDR20 (Fig. 1b). Specifically, Thr347 and Ser348 of USP46 form hydrogen bonds with Glu534 and Asn41 of WDR20, respectively; Glu279 and Asp293 of USP46 form salt bridges with Lys259 and Arg516 of WDR20, respectively. In addition, Val275 and Phe283 of USP46 make hydrophobic interactions with Trp306 and Phe262 of WDR20, respectively. Intriguingly, there is only a salt bridge interaction between Arg243 of WDR48 and Asp386 of WDR20 via the side faces of their WDR domains (Fig. 1c). Sequence alignments show that the residues of WDR20 involved in the interactions with USP46 are strictly conserved in different species (Supplementary Fig. S4a), and the residues of USP46 involved in the interactions with WDR20 are unvaried in USP12 but not conserved in USP1, which may explain why USP12 can bind to WDR20 but USP1 cannot (Supplementary Fig. S4b). These results suggest that the residues involved in the USP46-WDR20 interaction play an important role in the assembly of the complex and the regulation of the USP46 activity.

**Fig. 1:**
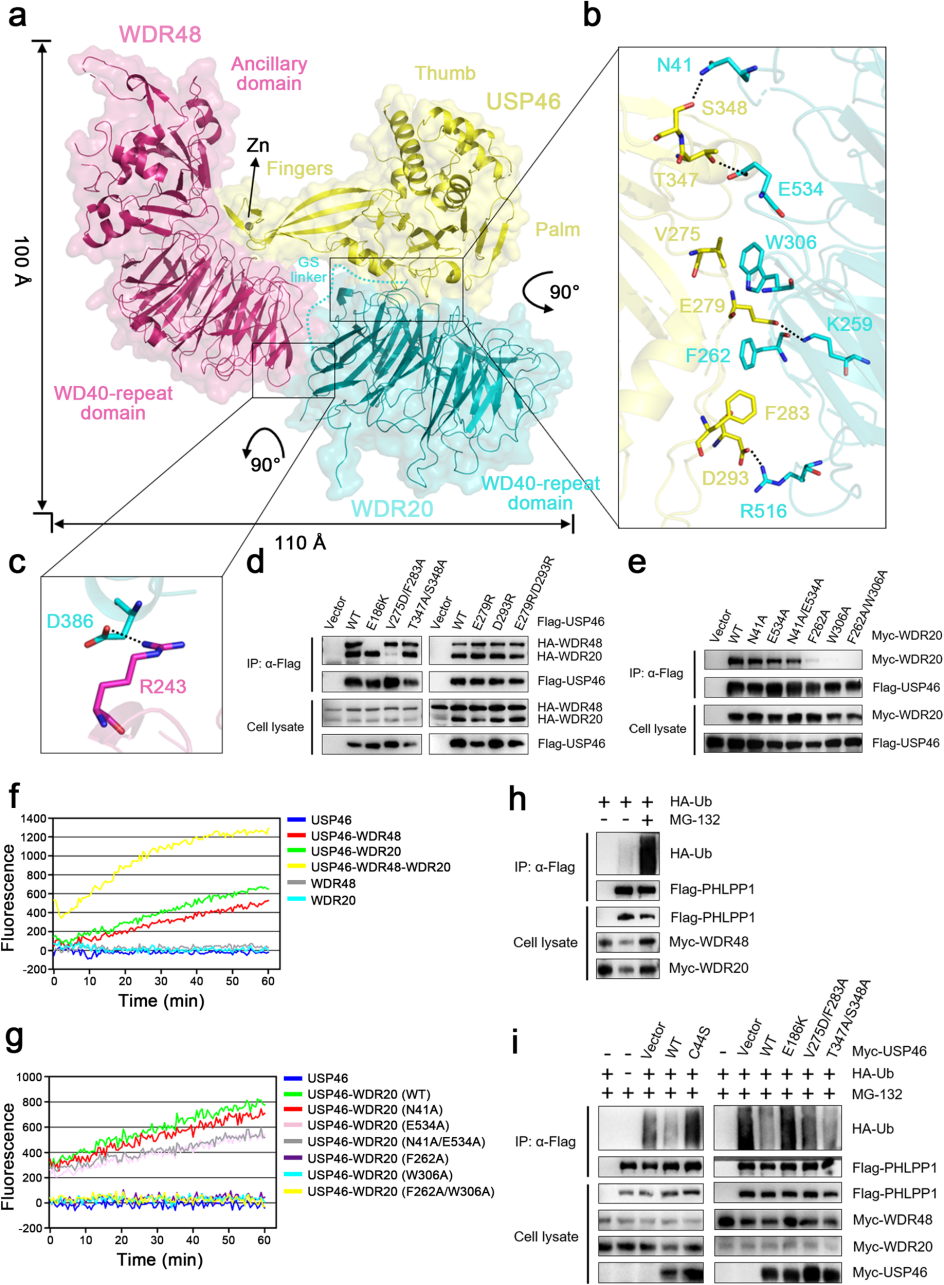
Structure and functional validation of the USP46-WDR48-WDR20 complex. **a** Overall structure of the USP46-WDR48-WDR20 complex. The complex is shown as a ribbon model in a transparent envelope surface, with USP46, WDR48, and WDR20 colored in yellow, pink and cyan, respectively, and the Zn^2+^ shown as a gray sphere. **b** Interaction interface between USP46 and WDR20. **c** Interaction interface between WDR48 and WDR20. The key residues involved in the interactions are shown with side chains in stick models. The hydrogen-bonding and salt-bridging interactions are indicated with dashed lines. **d**, **e** Co-IP assay to validate (d) the interaction of WT and mutant USP46 with WDR20 and WDR48 and (e) the interaction of WT and mutant WDR20 with USP46. **f**, **g** In vitro deubiquitination assay to verify the functional roles of the binding of (f) WDR20 and/or WDR48 and (g) the WDR20 mutants in the activation of USP46. **h**, **i** In vivo deubiquitination assay to examine the ubiquitination level of PHLPP1 in the presence of (h) WDR48 and WDR20 and (i) USP46, WDR48, and WDR20. The cell lysates were immunoprecipitated with the Flag or Myc antibodies and the ubiquitination level of PHLPP1 was detected using the HA antibody

To verify the biological relevance of the complex, firstly, we performed mutagenesis and Co-IP assay to examine the roles of the key residues at the USP46-WDR20 interface in the assembly of the complex in 293T cells. As positive and negative controls, WT USP46 can efficiently pull-down WDR48 and WDR20, and the E186K USP46 mutant which cannot interact with WDR48^12^, exhibits no binding with WDR48 but maintains binding with WDR20 (Fig. 1d). Mutation of the residues of USP46 involved in the hydrophobic interactions with WDR20 (V275D/F283A) disrupts its binding with WDR20, whereas those involved in the hydrophilic interactions with WDR20 (T347A/S348A, E279R, D293R, and E279R/D293R) have insignificant effects on its binding with WDR20; and all of these mutations have no effect on its binding with WDR48 (Fig. 1d). Conversely, mutations of the residues of WDR20 involved in the hydrophilic interactions with USP46 (N41A, E534A, and N41A/E534A) also have insignificant impacts on its binding with USP46, whereas those involved in the hydrophobic interactions (F262A, W306A, and F262A/W306A) significantly impair or completely abolish its binding with USP46 (Fig. 1e). These results indicate that the hydrophobic interactions at the USP46-WDR20 interface play an important role while the hydrophilic interactions acts a minor role, and that WDR20 and WDR48 can bind to USP46 independently, and the WDR20-WDR48 interaction has an insignificant role in the formation of the complex.

Secondly, we performed in vitro deubiquitination assay using Ub-AMC as the substrate to verify the roles of the key residues in the activation of USP46. As expected, the free USP46 has no measurable activity; both the USP46-WDR48 and USP46-WDR20 complexes exhibit moderate activity; and the ternary complex exhibits a significantly higher activity than the binary complexes (Fig. 1f). Consistent with our Co-IP assay results, the WDR20 mutants containing mutations N41A, E534A, and N41A/E534A can potentiate the USP46 activity to similar levels as WT WDR20, while the WDR20 mutants containing mutations F262A, W306A, and F262A/W306A cannot potentiate the USP46 activity (Fig. 1g). These results indicate that the binding of WDR48 and WDR20 can activate the USP46 activity independently and synergistically.

Furthermore, we performed in vivo deubiquitination assay to examine whether mutations of the key residues of USP46 affect its function in PHLPP deubiquitination^10^. In the absence of USP46, but presence of WDR48 and WDR20, PHLPP1 exhibits a very low ubiquitination level in the absence of MG-132 but a high ubiquitination level in the presence of MG-132 (a proteasome inhibitor to prevent the degradation of ubiquitinated substrate) (Fig. 1h). In the presence of WDR48 and WDR20, WT USP46 exhibits a high activity toward PHLPP1 and thus decreases the ubiquitination level of PHLPP1 significantly, whereas the C44S USP46 dead mutant does not (Fig. 1i). Consistently, the E186K USP46 mutant which cannot interact with WDR48, has no effect on the ubiquitination level of PHLPP1 as WDR48 is necessary for not only the activation of USP46 but also the recruitment of substrate^9,10,12^. Similarly, the V275D/F283A USP46 mutant which has no interaction with WDR20, can only slightly decrease the ubiquitination level of PHLPP1, while the T347A/S348A USP46 mutant which maintains the interaction with WDR20, can significantly reduce the ubiquitination level of PHLPP1 (Fig. 1i). These results indicate that the binding of WDR48 and WDR20 promotes USP46-mediated PHLPP1 deubiquitination in vivo. Our functional assays demonstrate that the USP46-WDR48-WDR20 complex is biologically relevant, and the binding of WDR48 and WDR20 can activate the USP46 activity.

We further performed a detailed comparison and re-analysis of all available USP12/46 structures and revealed the conformational changes of key structural elements surrounding the catalytic center that are associated with the binding of WDR48, WDR20, and Ub (Supplementary Figs. S5–7, Table S2). The structural and functional data together provide new insights into the molecular mechanisms of the activation of USPs by the WDR proteins. The binding of WDR48 induces conformational changes and increases conformational flexibility of several structural elements surrounding the catalytic center, which would weaken the substrate binding; meanwhile, the C-terminal domains of WDR48 are involved in the binding of substrate, which would enhance the substrate binding and the combined effects render a moderate activation of USPs. The binding of WDR20 can stabilize the conformations of the structural elements surrounding the catalytic center and enhances the substrate binding, and hence potentiates the activity of USPs. When WDR48 and WDR20 bind to USP12/46 together, the WDR20 binding restores the WDR48 binding-induced conformational changes and stabilizes the conformations of the structural elements surrounding the catalytic center, and consequently the binding of WDR48 and WDR20 has a synergistic effect on the activation of USP12/46.

## Supplementary information

Supplementary Information.

## Supplementary information

**Supplementary Information** accompanies the paper at (10.1038/s41421-019-0102-1).
