# Supplementary material for: Structural insights into the activation of USP46 by WDR48 and WDR20
Source: Cell Discov. 2019 Jul 2;5:34. doi: 10.1038/s41421-019-0102-1 (PMC6796834; doi:10.1038/s41421-019-0102-1)
Supplement: Supplementary file 1 — Supplementary Information. [file 41421_2019_102_MOESM1_ESM.docx]

**Structural insights into the activation of USP46 by WDR48 and WDR20**

**Hanwen Zhu^1^, Tianlong Zhang^1^, Fang Wang^1^, Jun Yang^1^, and Jianping Ding^1,^***

^1^ State Key Laboratory of Molecular Biology, CAS Center for Excellence in Molecular Cell Science, Shanghai Institute of Biochemistry and Cell Biology, University of Chinese Academy of Sciences, Chinese Academy of Sciences, 320 Yue-Yang Road, Shanghai 200031, China

* Correspondence: Jianping Ding (E-mail: jpding@sibcb.ac.cn)

**Supplementary Information**

**Materials and Methods**

**Cloning**

The DNA fragments encoding USP46 (residues 25-366), WDR48 (residues 1-580), WDR20 (residues 1-569) were amplified by PCR from the cDNA library of human cells. USP46 was cloned into the pET-28a plasmid (Novagen) attached with a C-terminal His_6_ tag. WDR20 was cloned into the pET-28a plasmid attached with an N-terminal His_6_ tag, in which two regions (residues 319-362 and 395-508) were replaced by linker GGGGS and GGGGS_4_, respectively. WDR48 was cloned into the pFastBac1 plasmid (Invitrogen) attached with an N-terminal Flag-His_6_-TEV tag. Mutants were generated using the QuikChange^®^ Site-Directed Mutagenesis Kit (Strategene). All constructs were confirmed by DNA sequencing.

**Protein expression and purification**

For structural study and *in vitro* deubiquitination assay, USP46 and WDR20 were expressed in *E. coli* BL21 (DE3) Codon-Plus strain (Tiangen) and the transformed cells were grown in LB medium at 37°C until OD_600_ reached 0.8 and then induced with 0.2 mM IPTG at 16°C for 20 h. WDR48 was expressed using the pFastBac baculovirus system in Sf9 insect cells (Invitrogen) for 48 h at 27°C after viral infection. The cells were harvested by centrifugation, resuspended in a lysis buffer (20 mM Tris-HCl, pH 8.0, 200 mM NaCl, and 1 mM PMSF), and then lysed by sonication. The precipitates were removed by centrifugation and the target proteins were purified by affinity chromatography using a Ni-NTA column (Qiagen) followed by size exclusion chromatography (SEC) using a Superdex 200 10/300 column (GE Healthcare) pre-equilibrated with a storage buffer (20 mM Tris-HCl, pH 8.0, 100 mM NaCl, and 5 mM DTT). To obtain the USP46-WDR48-WDR20 complex, the purified USP46, WDR48, and WDR20 were incubated together at 1:1:1 molar ratio at 4°C overnight and then purified by SEC using a Superdex 200 10/300 column pre-equilibrated with the storage buffer. The peak fractions were collected and then concentrated by ultrafiltration to 13.5 mg ml^-1^. The purified USP46-WDR48-WDR20 complex exists as a monomer in solution with high purity and homogeneity as shown by gel filtration and SDS-PAGE analyses (Supplementary Fig. S1).

**Crystallization and structure determination**

Crystallization of the USP46-WDR48-WDR20 complex was performed using the hanging drop vapor diffusion method by mixing equal volumes (1 μl) of the protein solution (about 13.5 mg ml^-1^) and the reservoir solution at 16°C. Crystals were obtained from drops consisting of a reservoir solution of 1.0 M NaH_2_PO_4_/K_2_HPO_4_ (pH 7.2). For diffraction data collection, the crystals were soaked in the reservoir solution supplemented with 30% glycerol and then flash-frozen in liquid nitrogen. Diffraction data were collected at 100 K at BL17U1 of Shanghai Synchrotron Radiation Facility, and were processed, integrated, and scaled together with HKL3000 (ref. ^1^). Statistics of the diffraction data are summarized in Table S1.

The structure of the USP46-WDR48-WDR20 complex was solved by the molecular replacement (MR) method as implemented in Phenix^2^ using the structure of the USP12-WDR48-WDR20 complex (PDB code: 5K1C)^3^ as the search model. Structure refinement was performed using Phenix and Refmac5 (ref. ^2,4^). Model building was carried out using Coot^5^. Structural analyses were carried out using programs in CCP4 suite^6^ and the PISA server^7^. Structure figures were generated using PyMol^8^. Statistics of the structure refinement and the final structure model are summarized in Table S1.

**Co-immunoprecipitation assay**

HEK293T cells (Invitrogen) were maintained in DMEM (Hyclone) medium supplemented with 10% fetal bovine serum (Biochrom). All cDNA fragments were cloned into the pcDNA 3 vector. Cells were transfected with various plasmids using Lipofectamine 2000 reagent (Invitrogen). For validation of the interactions in the USP46-WDR48-WDR20 complex, the cells were transfected with the following plasmids: Flag-USP46 and Myc-WDR20 (WT or mutants), Flag-USP46 (WT or mutants), HA-WDR48, and HA-WDR20.

For immunoprecipitation, 48 h after transfection, the cells were collected by centrifugation and lysed in a lysis buffer (50 mM Tris-HCl, pH 7.5, 120 mM NaCl, 1 mM EDTA, and 1% NP-40) supplemented with EDTA-free protease inhibitor (Roche) for 30 min at 4°C. The cell lysates were centrifuged at 16,000 g for 45 min, and then the supernatants were mixed with 25 μl anti-Flag M2 affinity gel (Sigma-Aldrich) pre-equilibrated with the lysis buffer at 4°C for 2.5 h. The gel was rinsed three times with the lysis buffer. Aliquots of the cell lysates and the immunoprecipitated proteins were resolved on SDS-PAGE followed by Western blot using monoclonal antibodies specific to Flag (Sigma-Aldrich; 1:3000), Myc (Sigma-Aldrich; 1:3000), and HA (Sigma-Aldrich; 1:3000).

***In vitro* deubiquitination assay**

*In vitro* deubiquitination activity assay was performed using Ub-AMC (Ub-7-amido-4-methylcoumarin; Boston Biochem) as the substrate. The purified proteins were diluted in reaction buffer (20 mM HEPES, pH 7.8, 20 mM NaCl, 0.5 mM EDTA, 0.1 mg ml^-1^ ovalbumin, and 10 mM DTT) at a final concentration of 1-20 nM prior to addition of the substrate. The assay was carried out in 100 μl of reaction buffer at 25°C using 96-well 1/2 area black plates. The cleaved AMC was excited at 345 nm and the fluorescence emission was monitored at 445 nm using a Synergy Neo2 Hybrid Multi-Mode Reader (BioTek Instruments Inc.). Each experiment was performed in triplicate. The diagrams were prepared using program Graphpad Prism (Graphpad Software).

***In vivo* deubiquitination assay**

Previously, USP46 was shown to play a tumor suppressor role in colon cancer through promoting PHLPP deubiquitination and then inhibiting Akt signaling^9^, suggesting that PHLPP might be a substrate of USP46. To investigate the functional role of the USP46-WDR48-WDR20 complex *in vivo*, we performed cellular deubiquitination assay to examine whether mutations of the key residues involved in the interactions of USP46 with WDR48 and WDR20 affect the function of USP46 in the PHLPP deubiquitination in 293T cells. HEK293T cells were co-transfected with HA-ubiquitin, Flag-PHLPP1, Myc-WDR48, Myc-WDR20, and Myc-USP46 (WT or mutants). The cells were transfected using Lipofectamine 2000 reagent and then treated with 10 μM MG-132 (Sigma-Aldrich) for 8 h before collection. After 48 h of transfection, the cells were collected by centrifugation and lysed in a lysis buffer (50 mM Tris-HCl, pH 7.5, 120 mM NaCl, 1 mM EDTA, and 1% NP-40) supplemented with EDTA-free protease inhibitor for 30 min at 4°C. The supernatant was incubated with 25 μl anti-Flag M2 affinity gel pre-equilibrated with the lysis buffer at 4°C for 2.5 h. After rinsed three times with the lysis buffer, aliquots of the cell lysates and the immunoprecipitated proteins were subjected to immunoblotting analysis using antibodies the same as in Co-IP assay.

**Text**

**Conformational changes of USP12/46 upon the binding of WDR48 and WDR20**

USP12 and USP46 are the smallest USPs containing only a USP domain, which share 89% sequence identity, and both of them can bind with and be activated by WDR48 and WDR20. Previous and our structural studies show that WDR48 binds to the tip of the fingers subdomain and WDR20 binds to the base of the palm subdomain of USP46 or USP12 (Fig. 1a). As both WDR48 and WDR20 are located remotely from the catalytic center of the USPs resided at the interface of the thumb and palm subdomains, it is not clear how the binding of the WDR proteins can potentiate the activity of the USPs. Previously, structural comparison of the USP46-Ub and USP46-WDR48-Ub complexes shows that the binding of WDR48 does not induce notable structural changes at and surrounding the catalytic center of USP46 but the ancillary and C-terminal SUMO-like domains of WDR48 encircle the bound Ub, suggesting that WDR48 might activate USP46 through stabilization of the substrate binding^10^. However, structural comparison of the free USP12, the USP12-WDR48 complex, and the USP12-WDR48-WDR20 complex shows that the binding of WDR48 induces a series of structural and conformational changes surrounding the catalytic center of USP12 including the pinky finger (PK), the PK helix, the blocking loop 1 (BL1), BL2, and BL3, and the catalytic cleft (CC) loop, and the further binding of WDR20 overwrites these changes by restabilizing these structural elements, suggesting that WDR48 and WDR20 might activate USP12 through different yet unknown allosteric mechanisms^3^. To further investigate the molecular mechanism of the activation of USP12/46 by WDR48 and WDR20, we performed a detailed comparison and re-analysis of the available USP12 and USP46 structures (Supplementary Table S2).

Among the three USP46 and five USP12 structures reported so far, there are five representative structural states: the free USP12, the USP12-WDR48 complex (determined in both *C*2 and *F*222 space groups), the USP12/46-WDR48-WDR20 complexes, the USP46-Ub complex, and the USP12/46-WDR48-Ub complexes^3,10,11^. Comparison of these structures shows that the catalytic triad of USP12/46 all adopts a catalytically conducive conformation and undergoes no major conformational changes upon the binding of WDR48, WDR20 or Ub, suggesting that the binding of WDR48, WDR20 or Ub has no significant impact on the structure of the catalytic center of USP12/46 (Supplementary Figs. S5a-b, S6a-b).

Compared with the free USP12 structure, the WDR48 binding to the fingers subdomain of USP12 not only stabilizes the pinky finger (PF) from a partially disordered loop to a well-defined β-sheet, but also transforms the fingers subdomain from a “semi-closed” conformation to a “closed” conformation (Supplementary Fig. S5a, c and Table S2). Concurrently, it also induces a series of other conformational changes on USP12, including a major downward displacement of the PK helix and disordering of the PK loop and BL1 loop (Supplementary Fig. S5c, d and Table S2). In addition, several structural elements surrounding the catalytic center also undergo conformational changes in the USP12-WDR48 structure determined in the *C*2 space group, including the BL2 converting from an inward loop into an outward loop, the BL3 converting from a partially defined outward loop into a partially defined inward loop, and the CC loop becoming disordered (Supplementary Fig. S5e); however, these conformational changes do not occur in the USP12-WDR48 structure determined in the *F*222 space group (Supplementary Fig. S5f). These results indicate that the WDR48 binding induces conformational changes of the pinky finger and the PK helix, and increases the conformational flexibility of the PK, BL1, BL2, BL3, and CC loops.

Comparison of the Ub-bound USP12/46 structures with the free USP12 and the USP12-WDR48 structures shows that in all the Ub-bound USP12/46 structures, the Ub is bound in the cleft formed by the fingers, palm, and thumb subdomains with its C-terminal tail extended into the catalytic center, and the pinky finger and the BL1, BL2, and CC loops are involved in interactions with the Ub^3,10,11^. In addition, most of the structural elements that undergo conformational changes upon the WDR48 binding, assume similar conformations as those in the free USP12 structure, except that the pinky finger assumes a β-sheet structure with a “semi-closed” conformation and the BL3 assumes a fully defined outward loop conformation (Supplementary Fig. S5c-e). These results indicate that the free USP12/46 is in a proper conformation for Ub binding; the Ub binding can stabilize the conformations of the structural elements surrounding the catalytic center and induces the pinky finger adopting a β-sheet structure with a “semi-closed” conformation and the BL1 loop adopting a β-sheet structure in order to make interactions with the Ub, but the WDR48 binding does not induce further conformational changes of USP12/46 in the presence of Ub. The stabilization of BL1 from a disordered conformation into a rigid β-sheet structure upon the Ub binding is also observed in the USP7 and USP18 structures^12,13^. The requirement for the BL1 to adopt a β-sheet structure for the Ub binding is also supported by the biochemical data showing that mutation of Phe262 on the BL1 of USP12 significantly impairs the activation of USP12 by WDR48 (ref. ^3^).

Since there is no USP12/46-WDR20 structure available, to dissect the impact of the WDR20 binding, we compared the USP46-WDR48-WDR20 complex with the free USP12, the USP46-Ub complex, and the USP12-WDR48 complex. Compared with the USP12-WDR48 complex, the further binding of WDR20 largely reverses the WDR48 binding-induced conformational changes of the PK helix and the PK, BL2, and CC loops back to their original conformations in the free USP12 structure (Supplementary Fig. S6a, c, d, f). Intriguingly, upon the WDR20 binding, the pinky finger assumes a well-defined β-sheet structure with an “extended” or “open” conformation (Supplementary Fig. S6d and Table S2); and the BL1 assumes a well-defined loop conformation (Supplementary Fig. S6e and Table S2). In addition, the BL3 assumes a loop conformation similar to that in the free USP12 but with a well-defined structure, and is involved in the interaction with WDR20 (Supplementary Fig. S6f). Moreover, a significant structural change occurs at the USP46-WDR20 interface. Specifically, a short β-sheet at the base of the fingers subdomain (residues 282-301) in the free and WDR48-bound USP12 (or residues 278-297 of USP46) is unwound into a loop (which is called the backhand (BH) loop) in the USP46-WDR48-WDR20 complex (Supplementary Fig. S6c). This structural change could be attributed to the π-π stacking interaction between Phe262 of WDR20 and Phe283 of USP46 (or Phe287 of USP12), which appears to pull Phe283 away from USP46 and thus disrupts the β-sheet conformation (Supplementary Fig. S6c). Meanwhile, to avoid steric conflict, the BH loop pushes the PK helix away by about 6 Å to adopt a conformation similar to that in the free USP12 (Supplementary Fig. S6c). Accompanying with the stabilization of the BL3 and the displacement of the PK helix, the PK, BL1, BL2, and CC loops are also stabilized in the conformations similar to those in the free USP12 (Supplementary Fig. S6d-f).

Intriguingly, compared with the USP12-WDR48 complex, the pinky finger in the USP12/46-WDR48-WDR20 complex is rotated downwards with the tip shifted by about 8 Å, transforming the fingers subdomain from a “closed” conformation into an “open” conformation (Supplementary Fig. S6d). This conformational change might be due to the interaction between WDR48 and WDR20, which appears to pull WDR48 towards WDR20 and further brings down the pinky finger (Fig. 1a, c). These results indicate that the further binding of WDR20 largely restores the WDR48-binding induced conformational changes and stabilizes the conformations of the structural elements surrounding the catalytic center. However, as the pinky finger with the “open” conformation and the BL1 with the loop conformation are not in proper conformations and positions to interact with the Ub, it is very likely that they will undergo conformational changes to adopt similar conformations as those observed in the Ub-bound USP12/46 structures, namely the pinky finger adopting a β-sheet structure with a “semi-closed” conformation and the BL1 with a β-sheet conformation.

**Molecular mechanism of the activation of USP12/46 by WDR48 and/or WDR20**

Based on the structural and functional results, we can propose the molecular mechanism of the activation of USP12/46 by WDR48 and/or WDR20. The free USP12/46 appears to have a weak ability to bind the Ub substrate. Although the BL1 loop assumes a β-sheet structure and is in a proper conformation and position to interact with Ub, the pinky finger assumes a partially disordered loop structure with a “semi-closed” conformation and is not in a proper conformation to interact with Ub. In addition, several structural elements surrounding the catalytic center including the PK helix, the PK, BL2, BL3, and CC loops appear to have inherently high flexibility. Thus, the Ub binding to the free USP12/46 is not tight or stable, and hence USP12/46 alone has no measurable activity^3,10,14-16^. This notion is consistent with the biochemical and structural data showing that the unmodified Ub cannot form a stable complex with USPs, and in all of the Ub-bound structures, the C-terminal Gly76 of Ub is modified to form a covalent bond with the catalytic Cys of USPs^10,11,13^.

The WDR48 binding to USP12/46 seems to have two fold effects. On one hand, the WDR48 binding to the fingers subdomain of USP12/46 induces conformational changes of the pinky finger and the PK helix, and increases the conformational flexibility of the PK, BL1, BL2, BL3, and CC loops, which would destabilize the substrate binding. On the other hand, the biochemical data show that the C-terminal SUMO-like domain of WDR48 is involved in the binding of substrates^9,17-19^, and the structural data also show that the ancillary and C-terminal SUMO-like domains of WDR48 encircle the bound Ub^10^, suggesting that the WDR48 binding would stabilize the substrate binding. It seems the positive impact surpasses the negative impact on the substrate binding and thus the apparent effect of the WDR48 binding is a moderate activation of USP12/46. This notion is supported by the biochemical data showing that WDR48 alone can moderately potentiate the activity of USP12/46 towards Ub-AMC *in vitro* (Fig. 1f), but cannot potentiate the activity of USP12 towards a peptide substrate Leu-Arg-Gly-Gly (LRGG)-AMC^3^, indicating that WDR48 is involved in the binding of the Ub substrate which leads to the activation of the USP12/46 activity.

Our biochemical data show that WDR20 alone can moderately potentiate the activity of USP46 towards Ub-AMC *in vitro* to a level slightly higher than WDR48 alone (Fig. 1f), suggesting that WDR20 alone can bind to USP12/46 and activate the activity of USP12/46. Since there is no USP12/46-WDR20 structure available, we can speculate the activation mechanism of USP12/46 by WDR20 as follows: the binding of WDR20 to the base of the palm subdomain of USP12/46 would stabilize the conformations of the BL3 and BH loops and subsequently the structural elements surrounding the catalytic center including the PK, BL1, BL2, and CC loops. As a result, the binding of WDR20 would enhance the substrate binding and thus potentiate the activity of USP12/46. It should be noted that in the putative USP12/46-WDR20 structure, the pinky finger and the BL1 may or may not assume similar conformations as those in the free USP12 or the USP12/46-WDR48-WDR20 complex; however, upon the substrate binding, they would undergo conformational changes and assume similar conformations as those in the USP12/46-WDR48-Ub complex.

When WDR48 and WDR20 bind to USP12/46 together, the WDR20 binding restores the WDR48 binding-induced conformational changes and stabilizes the conformations of the structural elements surrounding the catalytic center, and thus the binding of WDR48 and WDR20 has a synergistic effect on the activation of USP12/46. Nevertheless, in the USP12/46-WDR48-WDR20 complex, the fingers subdomain assumes an “open” conformation and the BL1 adopts a loop conformation, both of which are not in proper conformations and positions to interact with the Ub substrate. In particular, Phe258 of the BL1 in USP46 (or Phe262 in USP12) would be in steric conflict with Leu73 of the Ub substrate (Supplementary Fig. S7a)^10,11^. Thus, upon the substrate binding, these two structural elements must undergo conformational changes and assume similar conformations as those in the USP12/46-WDR48-Ub complex. Moreover, accompanying with the closure of the fingers subdomain, WDR48 would break its interaction with WDR20 and moves upwards along with the pinky finger to maintain its interactions with the pinky finger and to allow its C-terminal domains to interact with the Ub substrate (Supplementary Fig. S7b).

| **Supplementary Table S1. Data collection and refinement statistics** | |
| --- | --- |
| **Data collection** |  |
| Wavelength (Å) | 0.9791 |
| Space group | *P*6_1_22 |
| Cell parameters |  |
| *a*, *b*, *c* (Å) | 217.2, 217.2, 223.9 |
| *α*, *β*, *γ* (°) | 90, 90, 120 |
| Resolution (Å) | 50.0-3.10 (3.21-3.10)^a^ |
| Observed reflections | 616,763 |
| Unique reflections (I/σ(I) > 0) | 56,304 |
| Average redundancy | 11.0 (11.1) |
| Average I/σ(I) | 29.8 (2.3) |
| Completeness (%) | 99.0 (99.9) |
| *R*_merge_ (%)^b^ | 14.1 (180.0) |
| CC_1/2_ | 0.995 (0.519) |
| **Refinement and structure model** |  |
| No. of reflections (*Fo>*0σ(*Fo*)) | 56,224 |
| Working set | 53,401 |
| Test set | 2,823 |
| *R*-factor/ free *R*-factor (%)^c^ | 24.7/27.3 |
| No. of atoms | 9,327 |
| Protein | 9,273 |
| Ligand/ion | 54 |
| Wilson B-factor (Å^2^) | 88.1 |
| Average B-factor (Å^2^) | 95.0 |
| Protein | 95.0 |
| Ligand/ion | 93.7 |
| RMS deviations |  |
| Bond lengths (Å) | 0.002 |
| Bond angles (^o^) | 0.466 |
| Ramachandran plot (%) |  |
| Favored | 95.0 |
| Allowed | 5.0 |
| Outliers | 0.0 |
| ^a^ Numbers in parentheses represent the highest resolution shell.  ^b^ *[R](http://www.sciencedirect.com/science?_ob=MathURL&_method=retrieve&_udi=B6T9R-4S2VM65-1&_mathId=mml1&_user=7037937&_cdi=5121&_rdoc=1&_acct=C000054345&_version=1&_userid=7037937&md5=fc76e9c4e18e9cdf77302984378929dc)*_[merge](http://www.sciencedirect.com/science?_ob=MathURL&_method=retrieve&_udi=B6T9R-4S2VM65-1&_mathId=mml1&_user=7037937&_cdi=5121&_rdoc=1&_acct=C000054345&_version=1&_userid=7037937&md5=fc76e9c4e18e9cdf77302984378929dc)_ [= ∑*_hkl_*∑*_i_*\|*I_i_*(*hkl*)−<*I*(*hkl*)>\|/∑*_hkl_*∑*_i_I_i_*(*hkl*)](http://www.sciencedirect.com/science?_ob=MathURL&_method=retrieve&_udi=B6T9R-4S2VM65-1&_mathId=mml1&_user=7037937&_cdi=5121&_rdoc=1&_acct=C000054345&_version=1&_userid=7037937&md5=fc76e9c4e18e9cdf77302984378929dc).  ^c^ [*R*-factor = ∑*_hkl_*\|\|*F_o_*\|−\|*F_c_*\|\|/∑*_hkl_*\|*F_o_*\|](http://www.sciencedirect.com/science?_ob=MathURL&_method=retrieve&_udi=B6T9R-4S2VM65-1&_mathId=mml2&_user=7037937&_cdi=5121&_rdoc=1&_acct=C000054345&_version=1&_userid=7037937&md5=3fed7a56c99698e8d27159f8bf0cc858). | |

| **Supplementary Table S2. Summary of the conformations of key structural elements in different USP12 and USP46 structures** | | | | | |
| --- | --- | --- | --- | --- | --- |
| Structure | Fingers | Pinky finger | PK helix | PK loop | BL1 |
| USP46-Ub | semi-closed | β-sheet | -- | loop | β-sheet |
| USP46-WDR48-Ub | semi-closed | β-sheet | -- | loop | β-sheet |
| USP46-WDR48-WDR20 | open | β-sheet | -- | loop | loop |
| USP12 | semi-closed | partially disordered loop | -- | loop | β-sheet |
| USP12-WDR48 (*C*2) | closed | β-sheet | downward | disordered | disordered |
| USP12-WDR48 (*F*222) | closed | β-sheet | downward | disordered | disordered |
| USP12-WDR48-Ub | semi-closed | β-sheet | -- | loop | β-sheet |
| USP12-WDR48-WDR20 | open | β-sheet | -- | loop | loop |
| ***Continued*** |  |  |  |  |  |
| Structure | BL2 | BL3 | CC loop | BH loop | Reference |
| USP46-Ub | loop/inward | loop/outward | loop | β-sheet | [10] |
| USP46-WDR48-Ub | loop/inward | loop/outward | loop | β-sheet | [10] |
| USP46-WDR48-WDR20 | loop/inward | loop/outward | loop | loop | This work |
| USP12 | loop/inward | partially disordered loop/outward | loop | β-sheet | [3] |
| USP12-WDR48 (*C*2) | loop/outward | partially disordered loop/inward | disordered | β-sheet | [3] |
| USP12-WDR48 (*F*222) | loop/inward | partially disordered loop/outward | loop | β-sheet | [3] |
| USP12-WDR48-Ub | loop/inward | loop/outward | loop | β-sheet | [11] |
| USP12-WDR48-WDR20 | loop/inward | loop/outward | loop | loop | [3] |

**Supplementary Figure S1**

**
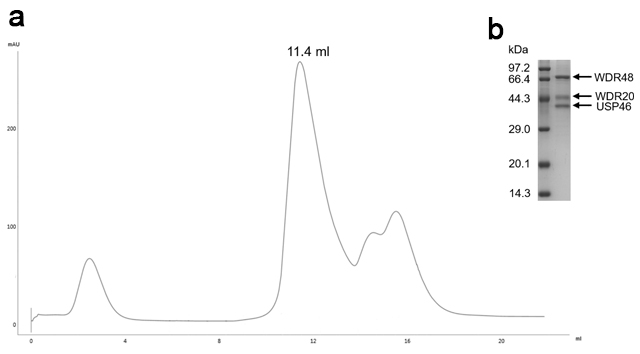
**

**Supplementary Figure S1. Size exclusion chromatography and SDS-PAGE analyses of the USP46-WDR48-WDR20 complex. (a)** Purification of the USP46-WDR48-WDR20 complex using size exclusion chromatography (SEC) on a Superdex 200 10/300 column. The peak fractions at the indicated elution volume (11.4 ml) were pooled for further structural and functional studies. **(b)** SDS-PAGE analysis of the purified USP46-WDR48-WDR20 complex. The peak fractions of the USP46-WDR48-WDR20 complex were resolved on SDS-PAGE followed by Coomassie-blue staining.

**Supplementary Figure S2**


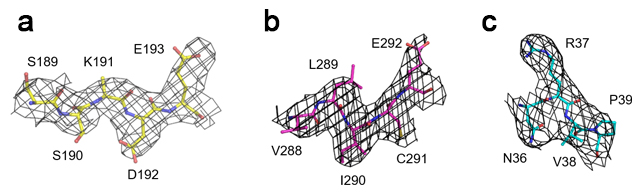


**Supplementary Figure S2. Representative simulated annealing composite omit map of the USP46-WDR48-WDR20 complex.** Simulated annealing composite omit map of **(a)** USP46, **(b)** WDR48, and **(c)** WDR20. Map is contoured at 1.5σ level with the final structure shown in ball-and-stick model. USP46, WDR48, and WDR20 are colored in yellow, pink, and cyan, respectively.

**Supplementary Figure S3**


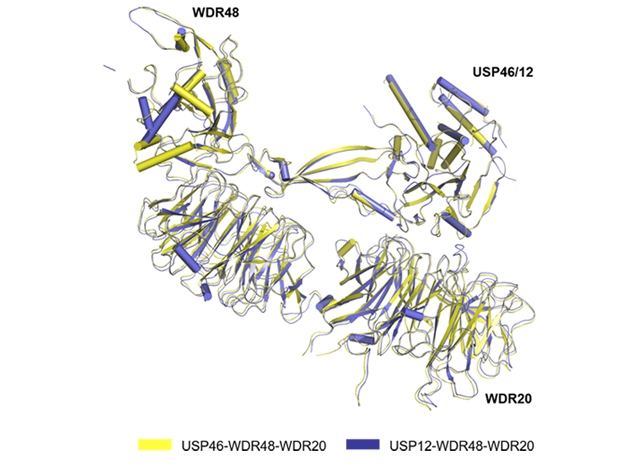


**Supplementary Figure S3. Structural comparison of the USP46-WDR48-WDR20 complex and the USP12-WDR48-WDR20 complex.** Superposition of the USP46-WDR48-WDR20 complex (colored in yellow) and the USP12-WDR48-WDR20 complex (colored in slate-blue) yields an RMSD of 0.49 Å for 1190 Cα atoms. Both complexes are shown in ribbon models.

**Supplementary Figure S4**


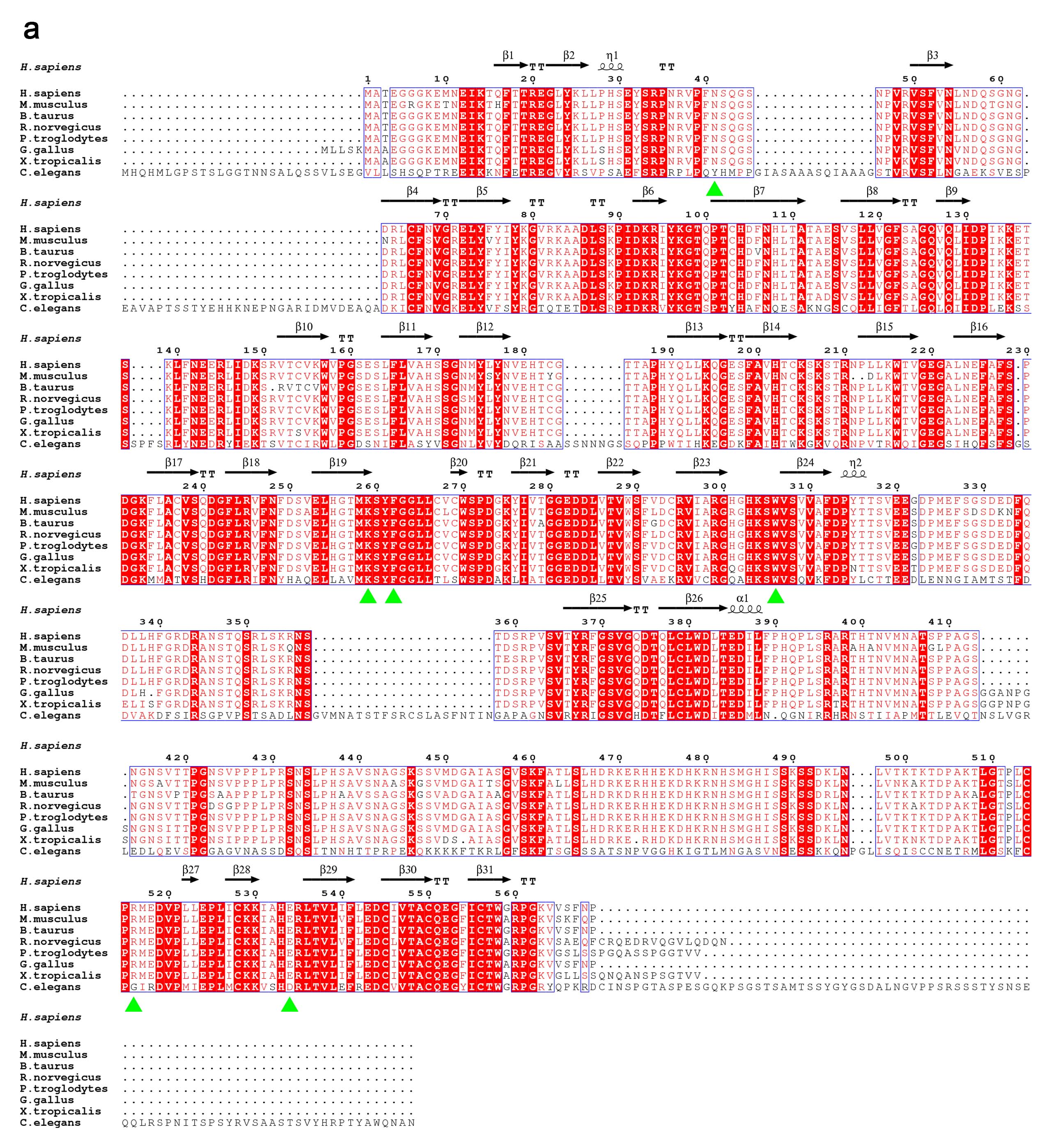


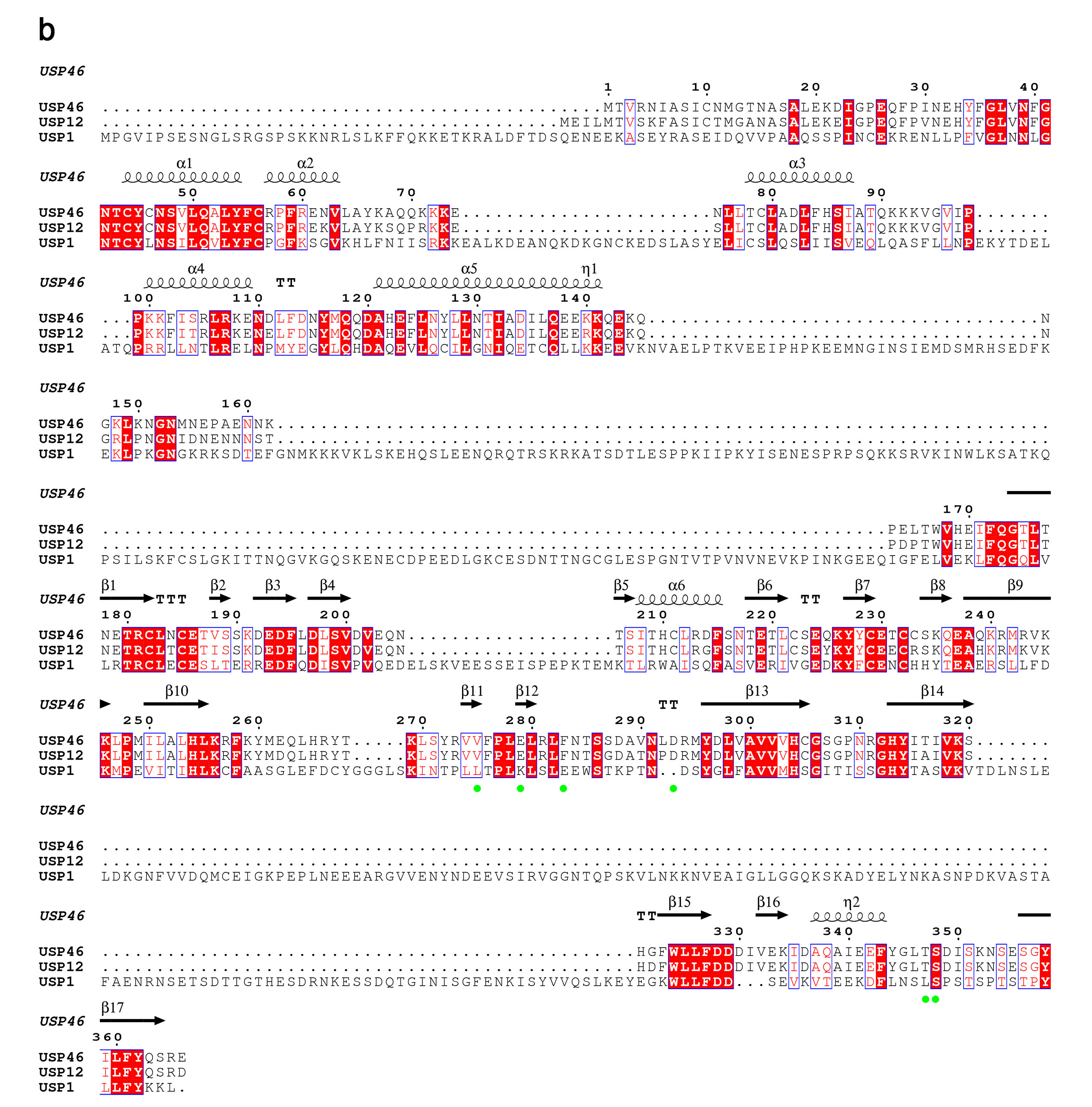


**Supplementary Figure S4. Sequence alignment of WDR20 from different species and USP46 with its homologs USP12 and USP1. (a)** The secondary structures of human WDR20 in the USP46-WDR48-WDR20 complex are placed on the top of the alignment. Shaded and open red boxes indicate identical and highly similar residues, respectively. The key residues involved in the interactions with USP46 are indicated with green triangles below. Species include: *Homo sapiens*, *Mus musculus*, *Bos taurus*, *Rattus norvegicus*, *Pan troglodytes*, *Gallus gallus*, *Xenopus tropicalis*, and *Caenorhabditis elegans*. **(b)** The secondary structures of USP46 in the USP46-WDR48-WDR20 complex are placed on the top of the alignment. The key residues involved in the interactions with WDR20 are indicated with green dots below.

**Supplementary Figure S5**


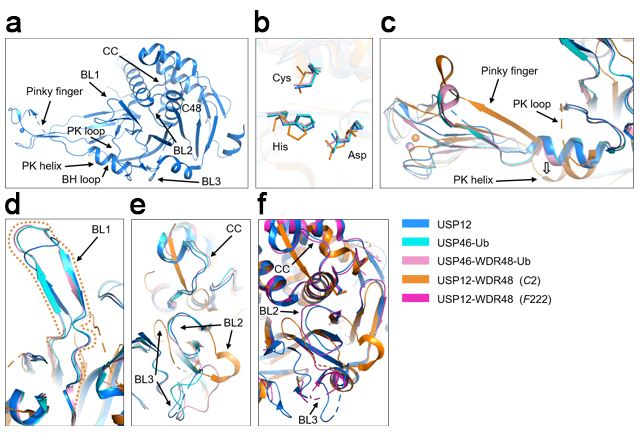


**Supplementary Figure S5. Conformational changes of the key structural elements in USP46/12 upon the binding of WDR48. (a)** Overview of the key structural elements surrounding the catalytic center and involved in the binding of the Ub substrate in the free USP12 structure (PDB code: 5K16). USP12 is shown in a ribbon model and colored in marine. **(b-e)** Structural comparison of USP12/46 in the free USP12 (colored in marine), the USP46-Ub complex (colored in cyan, PDB code: 5CVM), the USP46-WDR48-Ub complex (colored in pink, PDB code: 5CVN), and the USP12-WDR48 complex (determined in *C*2 space group, colored in orange, PDB code: 5K1A) reveals the conformational changes of the key structural elements of USP12/46 upon the binding of WDR48 and Ub, including **(b)** the catalytic triad, **(c)** the pinky finger, the PK helix, and the PK loop, **(d)** the BL1 loop, and **(e)** the BL2, BL3, and CC loops. **(f)** Superposition of the free USP12 and the USP12-WDR48 complex determined in the *C*2 space group and the *F*222 space group (colored in magenta, PDB code: 5K1B).

**Supplementary Figure S6**


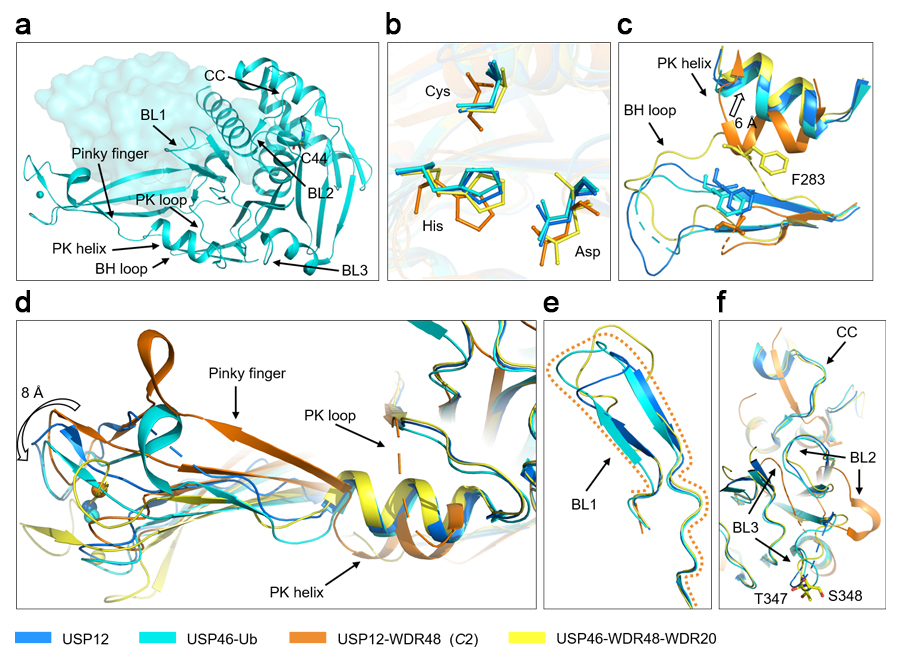


**Supplementary Figure S6. Conformational changes of the key structural elements in USP46/12 upon the binding of WDR48 and WDR20. (a)** Overview of the key structural elements surrounding the catalytic center and involved in the binding of the Ub substrate in the structure of the USP46-Ub complex (PDB code: 5CVM). USP46 and Ub are shown as a ribbon model and a transparent envelope surface colored in cyan, respectively. The catalytic residue Cys44 is shown as a stick model, and the Zn^2+^ as a sphere. **(b-f)** Structural comparison of USP12/46 in the USP46-WDR48-WDR20 complex (colored in yellow, this work), the free USP12 (colored in marine, PDB code: 5K16), the USP46-Ub complex (colored in cyan, PDB code: 5CVM), and the USP12-WDR48 complex (determined in *C*2 space group, colored in orange, PDB code: 5K1A) complex reveals the conformational changes of the key structural elements of USP12/46 upon the binding of WDR48, WDR20, and Ub, including **(b)** the catalytic triad, **(c)** the PK helix and the BH loop, **(d)** the pinky finger, the PK helix, and the PK loop, **(e)** the BL1 loop, and **(f)** the BL2, BL3, and CC loops. The catalytic triad, Phe283 on the BH loop of USP46 (or Phe287 of USP12), and Thr347 and Ser348 on the BL3 loop of USP46 are shown in stick models.

**Supplementary Figure S7**


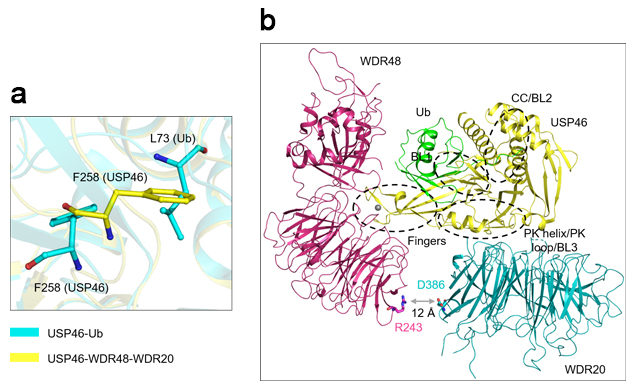


**Supplementary Figure S7. A structure model of the USP46-WDR48-WDR20-Ub complex. (a)** A zoom-in picture showing the potential steric conflict between Phe258 on the BL1 loop of USP46 and Leu73 on the tail of the Ub substrate based on superposition of USP46 in the USP46-WDR48-WDR20 complex (colored in yellow) and the USP46-Ub complex (colored in cyan, PDB code: 5CVM). **(b)** Overview of the structure model of the USP46-WDR48-WDR20-Ub complex. The model was generated based on the USP46-WDR48-WDR20 complex and the USP46-WDR48-Ub complex (PDB code: 5CVN). USP46, WDR48, WDR20 (colored as in Fig. 1a), and Ub (colored in green) are shown with ribbon models. Arg243 of WDR48 and Asp386 of WDR20 are shown in ball-and-stick models with their distance indicated. The key structural elements of USP46 that are predicted to undergo conformational changes upon the substrate binding are circled with black dashed lines.

**References**

1. Minor, W., Cymborowski, M., Otwinowski, Z. & Chruszcz, M. HKL-3000: the integration of data reduction and structure solution--from diffraction images to an initial model in minutes. *Acta Crystallogr. D Biol. Crystallogr.* **62**, 859-866 (2006).

2. Adams, P. D. et al. PHENIX: a comprehensive Python-based system for macromolecular structure solution. *Acta Crystallogr. D Biol. Crystallogr.* **66**, 213-221 (2010).

3. Li, H. et al. Allosteric activation of ubiquitin-specific proteases by β-propeller proteins UAF1 and WDR20. *Mol. Cell* **63**, 249-260 (2016).

4. Murshudov, G. N. et al. REFMAC5 for the refinement of macromolecular crystal structures. *Acta Crystallogr. D Biol. Crystallogr.* **67**, 355-367 (2011).

5. Emsley, P. & Cowtan, K. Coot: model-building tools for molecular graphics. *Acta Crystallogr. D Biol. Crystallogr.* **60**, 2126-2132 (2004).

6. Winn, M. D. et al. Overview of the CCP4 suite and current developments. *Acta Crystallogr. D Biol. Crystallogr.* **67**, 235-242 (2011).

7. Krissinel, E. & Henrick, K. Inference of macromolecular assemblies from crystalline state. *J. Mol. Biol.* **372**, 774-797 (2007).

8. Alexander, N., Woetzel, N. & Meiler, J. bcl::Cluster : a method for clustering biological molecules coupled with visualization in the Pymol Molecular Graphics System. *IEEE Int. Conf. Comput. Adv. Bio. Med. Sci.* **2011**, 13-18 (2011).

9. Li, X. et al. The deubiquitination enzyme USP46 functions as a tumor suppressor by controlling PHLPP-dependent attenuation of Akt signaling in colon cancer. *Oncogene* **32**, 471-478 (2013).

10. Yin, J. et al. Structural insights into WD-repeat 48 activation of ubiquitin-specific protease 46. *Structure* **23**, 2043-2054 (2015).

11. Dharadhar, S., Clerici, M., van Dijk, W. J., Fish, A. & Sixma, T. K. A conserved two-step binding for the UAF1 regulator to the USP12 deubiquitinating enzyme. *J. Struct. Biol.* **196**, 437-447 (2016).

12. Basters, A. et al. Structural basis of the specificity of USP18 toward ISG15. *Nat. Struct. Mol. Biol.* **24**, 270-278 (2017).

13. Hu, M. et al. Crystal structure of a UBP-family deubiquitinating enzyme in isolation and in complex with ubiquitin aldehyde. *Cell* **111**, 1041-1054 (2002).

14. Cohn, M. A. et al. A UAF1-containing multisubunit protein complex regulates the Fanconi anemia pathway. *Mol. Cell* **28**, 786-797 (2007).

15. Cohn, M. A., Kee, Y., Haas, W., Gygi, S. P. & D'Andrea, A. D. UAF1 is a subunit of multiple deubiquitinating enzyme complexes. *J. Biol. Chem.* **284**, 5343-5351 (2009).

16. Kee, Y. et al. WDR20 regulates activity of the USP12•UAF1 deubiquitinating enzyme complex. *J. Biol. Chem.* **285**, 11252-11257 (2010).

17. Yang, K. et al. Regulation of the Fanconi anemia pathway by a SUMO-like delivery network. *Genes Dev.* **25**, 1847-1858 (2011).

18. Lee, K. Y. et al. Human ELG1 regulates the level of ubiquitinated proliferating cell nuclear antigen (PCNA) through Its interactions with PCNA and USP1. *J. Biol. Chem.* **285**, 10362-10369 (2010).

19. Gangula, N. R. & Maddika, S. WD repeat protein WDR48 in complex with deubiquitinase USP12 suppresses Akt-dependent cell survival signaling by stabilizing PH domain leucine-rich repeat protein phosphatase 1 (PHLPP1). *J. Biol. Chem.* **288**, 34545-34554 (2013).
